# Culture density of menstrual blood-derived stromal/stem cells determines the quality of T cell responses: An experimental study

**DOI:** 10.18502/ijrm.v19i1.8182

**Published:** 2021-01-25

**Authors:** Shohreh Nikoo, Massoumeh Ebtekar, Mahmood Jeddi-Tehrani, Mahmood Bozorgmehr, Amir-Hassan Zarnani

**Affiliations:** ^1^Immunology Research Center, Iran University of Medical Sciences, Tehran, Iran.; ^2^Department of Immunology, Faculty of Medicine, Tarbiat Modares University, Tehran, Iran.; ^3^Monoclonal Antibody Research Center, Avicenna Research Institute, ACECR, Tehran, Iran.; ^4^Reproductive Immunology Research Center, Avicenna Research Institute, ACECR, Tehran, Iran.; ^5^Oncopathology Research Center, Iran University of Medical Sciences, Tehran, Iran.; ^6^Department of Immunology, School of Public Health, Tehran University of Medical Sciences, Tehran, Iran.

**Keywords:** Menstrual, Stromal cells, T cell response, Interferon-*γ*, Regulatory T cells, Cytokines.

## Abstract

**Background:**

Menstrual blood-derived stromal/stem cells (MenSCs) are a new population of refreshing and highly proliferative stem cells. Immunomodulatory effects of MenSCs profoundly depend on their relative density.

**Objective:**

To find whether MenSCs cultured at varying numbers would differentially affect the allogenic peripheral blood mononuclear cells (PBMCs) key features.

**Materials and Methods:**

PBMCs were co-cultured with various MenSCs numbers. PBMCs proliferation was investigated via ${}^{3}$H-thymidine incorporation. Flow cytometry was used to assess human leukocyte antigen (HLA)-DR, HLA-ABC, HLA-G, and co-stimulatory markers on MenSCs and the percentage of regulatory T cells (Tregs) among PBMCs. The concentration of cytokines was determined in supernatant of co-cultures.

**Results:**

The support of PBMCs proliferation at low MenSCs densities correlated with higher levels of pro-inflammatory interferon gamma (IFN-γ) in MenSCs/PBMCs co-culture and increased expression of HLA-DR by MenSCs. On the other hand, the suppressive property of MenSCs at higher densities was independent of Treg frequency, but correlated with a high concentration of Interleukin (IL)-6 and IL-10 in the co-cultures.

**Conclusion:**

Totally, at different seeding densities, MenSCs could differentially interact with PBMCs leading to significant changes in the level of anti- and/or pro-inflammatory factors. These preliminary in vitro results are suggested to be taken into consideration in experimental models of MenSC-based immunomodulation. Nonetheless, for efficient utilization of MenSCs anti-inflammatory features in pre-clinical disease models, we still need to broaden our knowledge on MenSC-immune system cross-talk; this could play a part in designing more optimized MenSCs injection modalities in the case of future pre-clinical and subsequently clinical settings.

## 1. Introduction

Mesenchymal stem cells (MSCs) can be recovered from different sources (1) and have been proposed to be beneficial for the treatment of such immunological complications as transplantation-induced graft versus host disease (GVHD) (2). Immunomodulation is the principle feature of almost all types of MSCs (3); indeed, MSCs have been demonstrated to suppress mixed lymphocyte reaction (MLR) (4), induce regulatory T cell (Treg) generation, and decrease T helper (Th)1 and Th17 differentiation (3, 5). However, MSC clinical utilization has been reported to be troublesome due to rather difficult and invasive accessibility and also limited proliferative capacity compared to certain stem cell subsets (6). As a comparatively novel and promising stem cell source, menstrual blood (MB) is a refreshing and easy-to-access MSC source; such cells have a high proliferation rate (7) and the capacity to self-renew and differentiate into cells of all three germ layers (8). In addition, regarding their immunophenotype, menstrual blood-derived stromal/stem cells (MenSCs) have been shown to share quite similar features with their conventional MSC counterparts (9).

However, data regarding MenSCs potential interaction with immune cells is scarce. In one study, MenSCs could suppress cellular proliferation in allogeneic MLR, hinder IFN-γ and TNF-α production, and stimulate Interleukin (IL)-4 generation (10); this was the first work suggesting MenSCs to share T cell inhibitory functions with conventional MSCs. We further demonstrated that, depending on their relative frequency, MenSCs exerted contrasting impacts on cell proliferation in MLR (7). Notably, similar dose-dependent immunomodulatory impacts have been reported for other MSCs (11, 12). We have also provided compelling data demonstrating that MenSCs inhibited optimal maturation of monocyte-derived dendritic cells (DCs) (13). Despite the very limited data on MenSC immunomodulatory impacts, they have been used to treat several immune-mediated disease models probably due to their similarities to the well-known MSCs (14, 15). Furthermore, several studies have compared the immunomodulatory features and therapeutic potential of MenSCs and MSCs. In particular, a recent study reported the lower immunosuppressive ability of MenSCs versus BM-MSCs; while MenSCs exerted no beneficial impacts in the experimental arthritis model, they were of a potential clinical use in a GVHD model (16).

Given the aforementioned advantages and no tumorigenicity and ethical issues associated with their medical applications, MenSCs could be viewed as potential tools besides typical MSCs for utilization in clinical settings (17). Nevertheless, there is a need to unravel the detailed mechanisms underlying the interaction between MenSCs and immune system cells.

Hence, this study explores whether or not the change in MenSCs number can affect the proliferation rate, cytokine level, and Treg frequency among PBMCs. Furthermore, considering the large fluctuations in IFN-γ concentration at different MenSCs-PBMCs ratios, the potential impact of this cytokine on the expression of human leukocyte antigen (HLA)-DR, HLA-ABC, HLA-G and co-stimulatory molecules by MenSCs was assessed. Information regarding the pattern, based on which the level of the aforementioned factors would fluctuate under various MenSCs concentrations, would pave the way for the establishment of improved and to-the-point MenSC inoculation strategies in the case of future pre-clinical disease models.

## 2. Materials and Methods

### Sample collection 

In this in vitro experimental study, MB specimens were collected from six apparently healthy individuals. All participants had normal menstrual cycles, with no history of vaginal infection three month prior to sample collection, were at reproductive age (20-40 yr old), had not received GnRH agonist or hormones for at least three months before sampling, and had no history of autoimmune diseases and/or malignancies. Donor enrolment was carried between 2016 and 2018 at the Avicenna Fertility Center (Tehran, Iran).

### Antibodies

FITC-conjugated monoclonal antibodies against CD80 and HLA-DR, as well as PE-conjugated monoclonal antibodies against CD86, CD40, CD45, and HLA-ABC were purchased from BD biosciences (San Jose, CA, USA). PE-conjugated anti-HLA-G antibody was purchased from Abcam (USA). Human Regulatory T Cell Staining Kit #3 (eBiosciences, USA) was used for detecting CD4, CD25, FOXP3 positive cells (Table I).

### Co-culture of MenSCs with allogeneic peripheral blood mononuclear cells (PBMCs)

The isolation of MenSCs and confirmation of their functional and immunophenotypic capacities were all carried out as per our previously published reports (8, 9). To assess the effect of MenSCs on proliferation of allogeneic PBMCs and regulatory T cells and cytokine secretion, an allogeneic MLR system was planned. PBMCs isolation was performed using Ficoll-Paque (Sigma, USA) density gradient centrifugation. MenSCs from passages two to four were treated with proliferation inhibitor, mitomycin C (Sigma, USA), and subsequently seeded in triplicate at various cell numbers beginning with 1 × 10${}^{5}$ cells/well of a 96-well flat-bottomed plate. Six hours later, allogeneic MLR was set up on MenSCs culture by adding 2 × 10${}^{5}$ PBMCs from two allogenic individuals. Culture was continued for three days in RPMI-1640 medium supplemented with 10% FBS and non-essential amino acids. In all experiments, the PBMCs were obtained from fixed two apparently normal volunteers (8). In order to examine the effect of cell-cell contact on Treg generation, an allogeneic MLR-MenSCs co-culture on transwell (0.4 µm pore size) culture system (Corning, New York, USA) was also set up. First, 2 × 10${}^{5}$ MenSCs were seeded into the lower chambers of 24 well transwell plates with RPMI-1640 and 10% FBS. Next, allogeneic PBMCs were loaded into the upper chambers (2 × 10${}^{5}$ from each individual/well). As the control group, allogeneic PBMCs were cultured in the absence of MenSCs (18).

### PBMCs proliferation assay

The MenSCs-mediated modulation of T cell proliferation in MLR system was assessed using ${}_{3}$H-thymidine incorporation. MenSCs were co-cultured with two populations of allogenic PBMCs for three days in a 5% CO${}_{2}$, humidified incubator. One micro Curie of ${}_{3}$H-thymidine (Amersham, Sweden) was subsequently added to each well with the incubation continuing for 18 hr. Proliferation was counted using a liquid scintillation counter (Wallac 1410, Pharmacia, Sweden) and results were reported as the mean of counts per minute (cpm) for each triplicate wells of the culture. As the control negative group, allogeneic PBMCs were cultured in the absence of MenSCs (named MLR cultures) (8).

### Analysis of CD4/CD25/FOXP3+ cells 

PBMCs were cultured with MenSCs both cell-cell contact and transwell modes for 72 hr as described in the previous section. Thereafter, PBMCs were harvested from the co-culture and stained using eBioscience${}^{\mathrm{TM}}$ Human Regulatory T Cell Staining Kit #3 for Treg detection as per manufacturer direction. In brief, the cells were labeled with CD4-FITC and CD25-PE or their isotype-matched control antibodies, washed using stain buffer (PBS with 2% fetal bovine serum [FBS]), fixed, permeabilized, and finally stained intra-cellularly with either PE.Cy5-conjugated anti-human FOXP3 Ab, or its isotype control Ab according to the manufacturer's instructions. Staining was measured using a Partec PAS III flow cytometer (Münster, Germany) and data were analyzed and prepared using the FlowJo software (version 7.6.1.). To measure the percentage of Tregs, CD4+ cells were selected in the lymphocytes gate. Then, CD25+FOXP3+ cells were measured among the CD4+ cells (8).

### Detection of HLA-DR expression on MenSCs 

Our previous results showed that MenSCs could suppress or support proliferation of PBMCs depending on MenSCs: PBMCs ratio (7). To explore the molecular mechanism behind this phenomenon, we used flow cytometry to explore the expression of HLA-DR on MenSCs added to allogeneic MLR system at low and high MenSC numbers. Cells were then harvested, washed in cold stain buffer, and incubated for 30 min in stain buffer containing FITC-HLA-DR and PE-CD45 antibodies. Afterward, cells were analyzed using a PAS III flow cytometer. Anti-CD45 was used to exclude PBMCs from HLA-DR analysis on MenSCs.

### Cytokine assay

Supernatants of the MenSCs/PBMCs co-cultures were collected after 72 hr and examined for the concentration of IFN-γ, IL-10, IL-6, IL-8, TNF-α, and IL-17A by sandwich ELISA using commercially available ELISA kits. All kits were purchased from BD (USA) except IL-17A ELISA kit which was from eBiosciences (USA). The minimal detection limits of the aforesaid cytokines were 4.7, 7.8, 3.1, 3.1, 7.8, and 7.8 pg/ml respectively.

### Treatment of MenSCs with IFN-γ

IFN-γ is one of the key cytokines involved in the modulation of immune responses. Indeed, we observed that in MenSCs/PBMCs co-cultures, the level of IFN-γ fluctuated in parallel with lymphocyte proliferation, Treg frequency, and HLA-DR expression. Thus, we sought to determine how the expression of major histocompatibility complex (MHC) and co-stimulatory molecules is affected by IFN-γ treatment. To this end, MenSCs were cultured without or with different concentrations of IFN-γ (BD, USA) ranging from 0.1 to 100 U/mL. The expression of HLA-DR, HLA-ABC, HLA-G, CD80, CD86, and CD40 was assessed by flow cytometry (19). In all flow cytometric experiments, the isotype-matched Abs were used to check for potential non-specific bindings (Table I).

**Table 1:** Antibodies used for MenSC staining


**Antibody name**	**Clone**	**µL of antibody/100 µL of staining buffer**	**Company**	**Cat #**
**PE mouse anti-human CD40**	5C3	20	BD	555589
**PE mouse anti-human CD45**	HI30	10	BD	555483
**FITC mouse anti-human CD80**	L307.4	20	BD	557226
**PE mouse anti-human CD86**	2331 (FUN-1)	20	BD	560957
**PE mouse anti-human HLA-ABC**	EMR8-5	5	BD	565291
**FITC mouse anti-human HLA-DR**	L243	10	BD	347403
**PE mouse anti-HLA-G antibody**	MEM-G/9	1	Abcam	ab24384
CD: Cluster of Differentiation, HLA: Human Leukocyte Antigen				

### Ethical considerations

Informed written consent was obtained from all participants before enrolling in this study. The study was approved by the Institutional Review Board and the Ethics Committee for Medical Research of Avicenna Research Institute (code: 88/592).

### Statistical analysis

Statistical comparisons were carried out via non-parametric Mann-Whitney test in GraphPad Prism version 5.0.4 for Windows, GraphPad Software, La Jolla California USA, www.graphpad.com, and data are expressed as median with range. In each section, data have been obtained from five independent experiments. P-values < 0.05 were considered as statistically significant.

## 3. Results

### MenSCs differentially affected lymphocyte proliferation depending on co-culture ratio 

**Figure 1:**
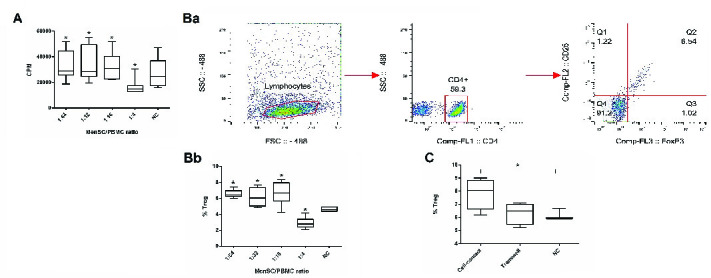
Impact of MenSCs on allogeneic MLR. Two allogeneic PBMCs were co-cultured in the presence of a third-party MenSCs at different ratios for three days and cell proliferation was measured by ${}^{3}$H-thymidine incorporation (A). Also, the percentage of Tregs was measured using flow cytometry; CD4+ cells were first gated in lymphocytes and then the percentage of CD25+FoxP3+ cells among CD4+ cells was measured (Ba) and compared between groups (Bb). The same culture system was also set up in a transwell culture plates with MenSCs and the two allogeneic PBMCs were seeded in the upper and lower chambers and Treg frequency was assessed as above (C). CPM: Count per minute; NC: Negative control (allogeneic MLR in the absence of MenSCs). P*-*values <0.05 are shown with*.

MenSCs at different ratios were added to a two-way allogeneic MLR culture system, and proliferation of PBMCs was then assessed through 3H thymidine incorporation. Accordingly, MenSCs significantly suppressed allogenic proliferation at the 1:4 ratio (MenSCs: PBMCs) compared to and the control group (without MenSCs) (p = 0.02). In contrast, at lower MenSCs numbers (1:16, 1:32, and 1:64 ratios), proliferation of PBMCs in allogeneic MLR system was significantly increased compared to control wells (p = 0.02) (Figure 1A).

### MenSCs-induced regulatory T cells expansion through cell contact-dependent mechanisms 

To investigate the Treg expansion by MenSCs, allogeneic PBMCs were co-cultured with MenSCs in either cell-cell contact or transwell culture systems for 72 hr. Thereafter, the T cells were analyzed for the expression of Treg markers using flow cytometry (Figure 1Ba). Our results showed that in the presence of cell-cell contact, the percentage of Tregs was decreased at 1:4 MenSCs-PBMCs ratio (p = 0.02); while at 1:16 to 1:64 ratios (lower MenSC numbers), the Treg population was significantly expanded (p = 0.03) (Figure 1Bb). On the contrary, in the transwell system (no cell-cell contact), MenSCs could not affect the percentage of Tregs (Figure 1C).

### MenSCs-modulated pro- and anti-inflammatory cytokine production in a dose-dependent manner

**Figure 2:**
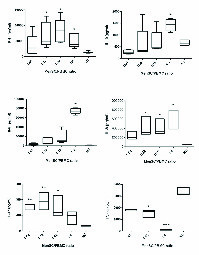
Effect of MenSCs on cytokine secretion in allogeneic MLR. Two allogeneic PBMCs were co-cultured in the presence of a third-party MenSCs at different ratios. After three days, the concentration of IFN-γ, IL-10, IL-6, IL-8, IL-17, and TNF-α was assessed in the supernatant using ELISA. NC: Negative control (allogeneic MLR without MenSCs). P-values < 0.05, 0.01, 0.001 are shown with *, **, and ***, respectively.

The impact of different numbers of MenSCs on the levels of IFN-γ, IL-10, IL-6, IL-8, IL-17, and TNF-α in two-way allogeneic MLR system was also investigated. To the results, IFN-γ, IL-17, IL-10, IL-6, and IL-8 level was significantly higher in the supernatant of MenSCs/PBMCs co-cultures in comparison to wells containing allogeneic PBMCs without MenSCs (control group) (p < 0.05-0.01). The TNF-α concentration was significantly lower at 1:4 (p = 0.001) and 1:16 (p = 0.02) ratios in the supernatant of MenSCs-PBMCs co-cultures. The number of MenSCs added to co-cultures positively correlated with its inhibitory impact on TNF-α production. Notably, lower numbers of MenSCs induced higher secretion of IFN-γ and IL-17, while it caused lower production of IL-10, IL-6, and IL-8 (Figure 2, Table II).

### IFN-γ-induced HLA-DR expression on MenSCs

**Figure 3:**
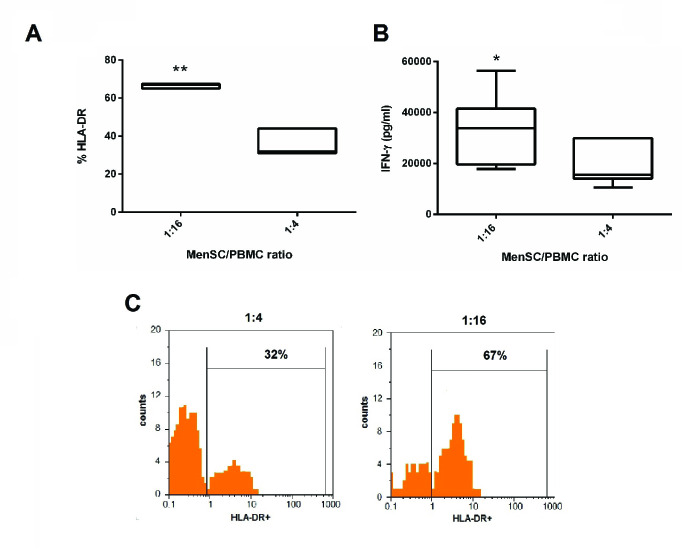
Evaluation of HLA-DR expression on MenSCs and IFN-γ production in the MenSCs/PBMCs co-culture. MenSCs were co-cultured with allogeneic PBMCs at two different ratios for three days. The expression of HLA-DR on CD45 negative cells (MenSCs) (A) and IFN-γ concentration in the cell culture supernatant was assessed by flow cytometry and ELISA (B), respectively. (C) Flow cytometry plots of a representative experiment regarding HLA-DR percentage on MenSCs. P-values < 0.05, and 0.01 are shown with *, and **, respectively.

**Figure 4:**
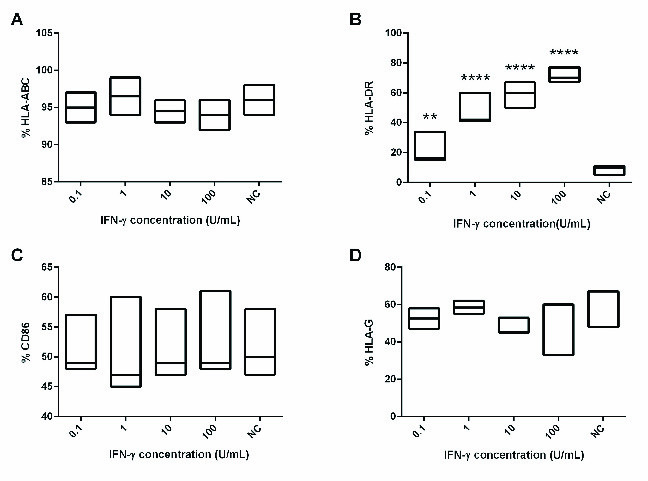
Effect IFN-γ treatment on HLA-ABC, HLA-DR, CD86, and HLA-G expression by MenSCs. MenSCs were treated with different concentrations of IFN-γ for two days and surface expression of the markers was assessed by flow cytometry. NC: Negative control (no IFN-γ treatment). P-values < 0.01 and 0.0001 are shown with **, and ****, respectively.

Previous studies have shown that MenSCs express HLA-ABC molecules but do not express HLA-DR at steady state (6). Hence, we next examined whether there is a relationship between IFN-γ and HLA-DR expression on MenSCs at different MenSCs-PBMCs ratios. With a decrease in the number of MenSCs in the co-cultures, both IFN-γ concentration and HLA-DR expression on MenSCs increased significantly (Figure 3). To further examine the impact of IFN-γ on HLA-DR expression on MenSCs, we treated MenSCs alone with varying concentrations of exogenous IFN-γ and compared the results with the steady state condition (no IFN-γ treatment). Nearly all MenSCs constitutively expressed HLA-ABC that was not affected by IFN-γ treatment (Figure 4A). However, HLA-DR that was only expressed by a minority of MenSCs at steady state showed a considerable upregulation with increasing IFN-γ concentration (p = 0.001) (Figure 4B). Furthermore, we found that MenSCs did not express co-stimulatory molecules, CD40 and CD80, but expressed moderate levels of CD86 (45%) at the steady state condition (Figure 4C). Importantly, there were no significant changes in the expression of CD40, CD80, and CD86 by MenSCs after treatment with different concentrations of IFN-γ. In addition, about 60% of MenSCs expressed HLA-G and activation with IFN-γ exerted no significant impacts on the expression level of this marker (Figure 4D).

**Table 2:** Cytokine concentration at various MenSC: PBMC ratios


**Cytokine**	**MenSC: PBMC ratio**				
	NC*	**1:4**	**1:16**	**1:32**	**1:64**
**IL-6**	1629 (331)	73976 (10042)	12077 (24884)	5028 (13690)	2980 (3354)
**IL-8**	47538 (2931)	777920 (410356)	336060 (324032)	311896 (393031)	203346 (170151)
**IL-10**	615 (231.6)	1432 (487)	481 (660.6)	333.3 (869)	269.3 (157.6)
**IL-17**	61.7 (9.7)	197.5 (163.1)	232 (249)	381 (222)	331.9 (66.2)
**IFN-γ**	5444 (3009)	15520 (17131)	33917 (27400)	18944 (29236)	11117 (20962)
**TNF-α**	3264 (625)	1839 (135)	1667 (643)	136.6 (74.6)	-
*Data are presented as median (interquartile range) MenSC: Menstrual blood-derived stromal/stem cells; PBMC: peripheral blood mononuclear cells; NC: Negative control					

## 4. Discussion

In the present study, we report novel aspects of MenSCs immunomodulatory mode of action. We had previously shown that the impact of MenSCs on PBMC proliferation could either be inhibitory or stimulatory depending on the MenSCs' number (7). Besides the injection route, injection interval, the nature of the disease model, and the source from which a type of MSC is obtained, one must also consider another key factor, that is, the number of injected cells. One of the preliminary steps in this respect is the establishment of set of in vitro co-cultures at different cell ratios between the cells being injected and its most probable target in the recipient; this might suggest some points regarding the potential impacts that the injected cell could exert as well as the underlying involved mechanisms (16, 18). Such results can further help the researcher to narrow down their choices regarding such considerations as the most efficient cell number to be injected and the injection intervals and finally spare a considerable amount of time, expense, and most importantly number of animals. Indeed, similar strategies have been used in the case of MSC usage for GVHD therapy as well as MenSC utilization for the treatment of two disease models (16, 18). Needless to say, the subsequent clinical steps would in part, if not thoroughly, be backed by data obtained from those preliminary in vitro studies. Accordingly, in this study, we have shown that there is a relationship between varying numbers of MenSCs in co-culture with PBMC and the concentration of several immunomodulatory cytokines, as well as the expression of co-stimulatory markers. This aspect has been addressed by two previously independent studies. In one study, the immunosuppressive capabilities of MenSCs and BM-MSCs in MLR system were investigated and compared.

Accordingly, when responder T cells were stimulated in the presence either MenSCs or BM-MSCs at a concentration of 10,000 cells per well, the BM-MSC were generally more immunosuppressive. However, at the concentration of 100,000 cells per well, MenSCs were significantly more immunosuppressive (20). The same trend was also reported in another study where MenSCs showed a similar suppressive impact on the proliferation of T cells at the 1:10 ratio in comparison with BM-MSCs. However, in contrast to BM-MSCs that maintained their inhibitory potential at a higher ratio (1:100), MenSCs had a stimulatory effect on T cell proliferation at higher ratios (16). Some other studies have shown the same trend for allogeneic PBMCs proliferation mediated by MSCs. Accordingly, Najar and colleagues showed that bone marrow-derived MSCs are able to either support or suppress T cell proliferation at low- and high densities, respectively. They proposed IL-6 as the key player responsible for the increase of T cell proliferation at lower densities of MSCs (11). In another study conducted by Fang and co-workers higher proliferative capacity of PBMCs in the presence of lower density of human MSCs was linked to increased levels of IL-2 and IFN-γ resulting in the MHC-II upregulation on MSCs (12). Similarly, our results showed that augmented T cell proliferation mediated by lower density of MenSCs is accompanied by higher concentrations of IFN-γ and a significant upregulation of HLA-DR on MenSCs. It is noteworthy that the enhanced PBMCs proliferation at lower MenSCs densities could not solely attributed to a few cytokines as the interaction of MenSCs with the cells of the immune system potentially results in the production of a vast array of cytokines and mediators with either contrasting or synergizing impacts. For instance, at higher MenSCs densities (numbers), where proliferation of PBMCs was inhibited, we found increased levels of IL-6 which is known to promote T cell proliferation (21), however, at the same time, levels of IL-10 was also increased which could inhibit proliferation of activated T cells (22). Indeed, it has been reported that IL-6 produced by human MSCs is, at least in part, responsible for the shift of M0 macrophages into IL-10-producing cells in vitro (23).

To our results, there was a positive correlation between MenSCs seeding density and the concentration of IL-6, implying the possible role of IL-6 in modulation of PBMC proliferative response. Our previous results also showed that MenSCs could efficiently prevent DCs from fulfilling their phenotypic maturation which was accompanied by high levels of IL-6 and IL-10 production (13). Furthermore, decreased levels of TNF-α and lower lymphocyte proliferation were detected in the presence of higher MenSC numbers. It has been shown that human MSCs caused mature DCs type 1 (DC1) to decrease TNF-α secretion and mature DC2 to increase IL-10 secretion (24). Moreover, quite convincing data published recently showed that IL-6, besides its pro-inflammatory role, could function as an anti-inflammatory cytokine through suppression of the secretion of many pro-inflammatory cytokines, such as IL-1, TNFα, GM-CSF, and IFNγ (25). We also observed MenSCs could increase the production of IL-17, which has also been reported for other sources of MSCs including those isolated from synovium and fetal bone marrow (26, 27).

Considering the opposite impacts of varying densities of MenSCs on the production of cytokines, we concluded that MenSCs could either promote or inhibit the production of pro- or anti-inflammatory responses depending on their relative density. MSCs have been reported to exert antigen-presenting activities under the tight control of IFN-γ. Such activity is accompanied by high expression level of MHC-II (19). In this regard, Desai and colleagues showed that MSCs caused a significant increase in the proliferation of antigen-challenged lymphocytes from allergic rhinitis subjects. They revealed that this correlated with increased production of inflammatory cytokines from T cells and increased expression of MHC-II and CD86 on MSCs (28). Our finding on the dual impact of MenSCs on lymphocyte proliferation could be explained, in part, by the dose-dependent modulation of APC function by MenSCs.

To further unravel the mechanisms responsible for the lymphocyte proliferation fluctuation in different MenSCs densities, the percentage of Treg cells was examined. Our results showed that during the 72-hr cell culture period, the relative frequency of Tregs was not changed in different MenSCs: PBMCs ratios indicating that changes in proliferation of PBMCs were independent of Tregs frequency. In fact, one of the reasons could be that to observe changes in Treg frequency, we might need culture times longer than 72 hr. Furthermore, we found that cell-cell contact is indispensable for Tregs expansion in the presence of MenSCs; this suggests that the production of the soluble factors needed for Treg expansion depends on the direct contact between the effector cells and MenSCs. In fact, several studies have shown that BM-MSCs cultured with stimulated PBMCs can induce the expansion of functional CD4+CD25highFOXP3+ Tregs (29, 30). Other sources of MSCs including those derived from human placenta and umbilical cord exhibited the same promoting action on Tregs (31, 32). A number of mechanisms have been suggested, both cell-contact-dependent and -independent mechanisms, however, there is no clear consensus as of yet.

The increased percentage of CD4+CD25+FOXP3+ Tregs and levels of pro-inflammatory cytokine at lower MenSC might indicate that Tregs are in part responsible for production of such cytokines. In this regard, it has been reported that during their activation in the presence of appropriate inflammatory stimuli in vitro, both human and murine Treg cells display an IL17+FOXP3+CD4+ phenotype and can produce IL-17 (33, 34). Preclinical studies also have demonstrated that the therapeutic effect of MenSCs on the course of experimental murine colitis is associated with significantly higher level of CD4+CD25+FOXP3+ Treg cells (15). In parallel, we showed that pretreatment of MenSCs with IFN-γ resulted in an upregulation of HLA-DR in a dose-dependent manner, a finding that had been reported earlier for BM-MSCs (19). Therefore, we hypothesize that by decreasing the MenSC density (number), it is the higher IFN-γ concentration and consequently higher HLA-DR expression that is, in part, responsible for higher proliferation of PBMCs. In line with this notion, it has been recently reported that the lower suppressive activity of MenSCs compared to BM-MSCs is associated with a significantly lower expression level of IFN-γ receptor-1 and -2 (16). Therefore, it seems that in contrast to BM-MSCs, the initiation of immunosuppressive machinery by MenSCs does not rely mainly on the IFN-γ signaling pathway. Interestingly, HLA-G protein expression on MenSCs was constitutive and the level did not alter in the presence of IFN-γ. In this regard, a study by Nasef and colleagues showed that MSCs constitutively express HLA-G, the level of which is not modified upon stimulation by allogeneic lymphocytes in the MSC/MLR co-culture (35).

## 5. Conclusion

MenSCs can collectively modulate immune responses; however, MenSCs number and consequently the dominant microenvironment components play key roles in presenting MenSCs as immune suppressor or enhancer cells. Therefore, we suggest that the researcher consider these variables while investigating MenSCs impacts on immune responses in vitro or preclinical settings.

##  Conflict of Interest

The authors declare that they have no potential conflict of interest.
